# *Besnoitia besnoiti* tachyzoite replication in bovine primary endothelial cells relies on host Niemann–Pick type C protein 1 for cholesterol acquisition

**DOI:** 10.3389/fvets.2024.1454855

**Published:** 2024-08-09

**Authors:** Camilo Larrazabal, Carlos Hermosilla, Anja Taubert, Liliana M. R. Silva

**Affiliations:** ^1^Institute of Parasitology, Biomedical Research Center Seltersberg, Justus Liebig University Giessen, Giessen, Germany; ^2^Department of Veterinary Sciences and Public Health, Universidad Católica de Temuco, Temuco, Chile; ^3^Egas Moniz Center for Interdisciplinary Research (CiiEM), Egas Moniz School of Health & Science, Caparica, Almada, Portugal; ^4^MED-Mediterranean Institute for Agriculture, Environment and Development & CHANGE-Global Change and Sustainability Institute, Universidade de Évora, Évora, Portugal

**Keywords:** *Besnoitia besnoiti*, Niemann–Pick type C protein, NPC1, U18666A, cholesterol

## Abstract

*Besnoitia besnoiti* is a cyst-forming apicomplexan parasite and the causal agent of bovine besnoitiosis. During early phase of infection, tachyzoites replicate within host endothelial cells in a host cell cholesterol-dependent process. By applying U18666A treatments, we here evaluated the role of Niemann–Pick type C protein 1 (NPC1) in both, intracellular *B. besnoiti* replication and host cellular cholesterol distribution. Additionally, *B. besnoiti*-driven changes in NPC1 gene transcription were studied by qPCR. Overall, U18666A treatments significantly reduced *B. besnoiti* proliferation and induced cholesterol accumulation in host cytoplasmic dense vesicles. However, NPC1 gene transcription was not affected by *B. besnoiti* infection.

## Introduction

*Besnoitia besnoiti* is an obligate intracellular apicomplexan parasite belonging to the cyst-forming group of coccidia, and represents the causative agent of bovine besnoitiosis, a cattle disease reemerging in Europe (1). In this context, clinical bovine besnoitiosis is provoked by tachyzoite proliferation and characterized by general symptoms, such as fever, lethargy, tachycardia, tachypnea, congested mucosae, edema, anorexia, and weight loss during acute infection phase, while the chronic phase is marked by bradyzoites cyst formation causing significant skin alterations and potential bull infertility (1, 2). Overall, during the febrile acute stage of besnoitiosis, tachyzoites primarily proliferate within endothelial cells of various organs and vessels, leading to vasculitis, thrombosis, and necrosis of venules and arterioles (1, 3, 4). In line, cumulated *in vitro* evidence has confirmed the suitability of primary bovine endothelial cell lines for *B. besnoiti* infection and proliferation, with replication rates similar to other coccidian parasites, such as *Toxoplasma gondii* or *Neospora caninum*, resulting in host cell lysis after 48 to 72 h (5–8), thereby effectively serving as an *in vitro* host cell model that closely mimics the *in vivo* scenario.

Like other coccidian parasites, intracellular replication of *B. besnoiti* is characterized by a rapid proliferation of tachyzoites (7, 8), therefore requiring a huge amount of cell building blocks. However, apicomplexans have undergone gene losses and consequently lack molecules essential for cholesterol biosynthesis (9). Therefore, to fulfil their molecular requirements, apicomplexan parasites employ diverse strategies, such as host organelle sequestration, host cell molecule scavenging and host gene modulation (10–12). Physiologically, cellular cholesterol can be acquired by both *de novo* biosynthesis and uptake from extracellular sources, with the latter significantly relying on low density lipoprotein (LDL)-based cholesterol endocytosis (13, 14). Mechanistically, plasmatic LDL particles are bound to LDL receptors (LDLR), and LDL-LDLR complexes are formed in a clathrin-dependent process, being internalized later (13, 14). This endocytic product undergoes hydrolysis within early endosomes being enriched with acid-containing vesicles, finally allowing LDLR recycling to the cytoplasmic membrane. Simultaneously, LDL-derived cholesterol is cleaved by acid lipase activity and thereafter incorporated as free cholesterol into the endoplasmic reticulum (ER) (13, 14). Notably, the hydrophobic nature of cholesterol requires its transport from late endosomes to the ER, a process facilitated by sterol carrier proteins (13, 14). In mammals, this transport is largely dependent on the Niemann–Pick type C protein 1 (NPC1). Cholesterol then is esterified and stored as cholesteryl esters together with other neutral lipids in cytoplasmic lipid droplets, which have been demonstrated to be scavenged by apicomplexan parasites (10, 11, 15, 16). In this context, the pivotal role of host NPC1 in cholesterol acquisition has been reported for other apicomplexans, such as *T. gondii*, *N. caninum*, *Plasmodium* spp., and *Eimeria bovis* (10, 11, 17, 18). Intriguingly, studies on *B. besnoiti* tachyzoite proliferation have revealed a dual participation of both LDL-driven and *de novo* biosynthesis-based cholesterol pathways (8), suggesting that *B. besnoiti* may differ from other fast proliferating apicomplexans in terms of cholesterol acquisition. Hence, the primary objective of this study was to assess the role of NPC1 in *B. besnoiti* replication in primary endothelial cells.

## Methods

### Primary host cell and *Besnoitia besnoiti* tachyzoites culture

Primary bovine umbilical vein endothelial cells (BUVEC) were used in this study given its suitability for infection and proliferation, and the *in vivo* endothelial tropism of *B. besnoiti* tachyzoites (4, 8). BUVEC were isolated according to established methods (19). Cells were maintained at 37°C in a 5% CO_2_ atmosphere in a modified endothelial cell growth medium (modECGM), composed by ECGM medium (PromoCell) diluted in 1:3 ratio with M199 (Sigma-Aldrich) and supplemented with 500 U/mL penicillin (Sigma-Aldrich), 50 μg/mL streptomycin (Sigma-Aldrich) and 5% fetal calf serum (FCS; Biochrom). As a primary cell type, only BUVEC isolates at less than three passages were utilized.

*B. besnoiti* tachyzoite stages (strain *Bb* Evora04) were propagated by successive passages in Madin Darby bovine kidney cells (MDBK) as described elsewhere (8). Cells were cultured in RPMI medium (Sigma-Aldrich), supplemented and maintained as described above. Vital parasites were harvested from infected host cell supernatants, pelleted (800 × g; 5 min) and re-suspended in modECGM for immediate further experiments.

### Inhibitor treatment

Pharmacological inhibition of NPC1 was achieved by the NPC1 blocker U18666A (20) (Cayman, diluted in DMSO; 23 mM). U18666A was diluted in modECGM at varying concentrations (0.93–7.5 μM), with confirmed non-cytotoxic effect on BUVEC as described elsewhere (18). Treatments were applied to fully confluent cell monolayers seeded on fibronectin-coated 12-well plates (Sarstedt; 1:400; Sigma-Aldrich) 48 h prior to infection. At this point, the inhibitor was removed, cells were washed to remove any traces of U18666A and were infected with *B. besnoiti* tachyzoites at a multiplicity of infection (MOI) of 1:5. The infection process lasted for 4 h under inhibitor-free conditions. Then, extracellular tachyzoites were removed and the inhibitor was added again to the cell culture medium. At 48 h *post infectionem* (p. i.), tachyzoites present in cell culture supernatants were collected, pelleted (800 × g; 5 min) and quantified using a Neubauer chamber. Diluted DMSO was included as vehicle control setting.

### Live cell 3D-holotomographic microscopy

To visualize the effects of U18666A treatments on parasite development and host cellular cholesterol distribution, BUVEC were seeded on 35 mm tissue culture μ-dishes (Ibidi^®^), treated with U18666A (7.5 μM) or vehicle (DMSO) and infected with *B. besnoiti* tachyzoites (MOI of 3:1). At 24 h p. i., 3D-holotomographic microscopy was performed using a 3D Cell-Explorer (Nanolive). Microscopic data were analyzed by STEVE software (Nanolive) to generate refractive index (RI)-based z-stacks. Thereafter, the average pixel intensity was estimated in the area surrounding each cell (defined as region of interest) from max-intensity projected RI maps by ImageJ software v1.54 (21), based on the correlation between RI and cell-dry matter content (22).

### Cholesterol visualization and quantification

Cellular free cholesterol distribution was visualized via filipin III staining [35 μg/mL; 1 h, 37°C; Cayman (23),] in *B. besnoiti*-infected cells and non-infected controls (seeded on 35 mm tissue culture μ-dishes, Ibidi), via epifluorescence using the 3D Cell-Explorer (Nanolive). Additionally, to determine the impact of U18666A on BUVEC neutral lipid content, cells were seeded into 25 cm^2^ flasks (Sarstedt) and treated with U18666A (7.5 μM) for 48 h. Thereafter, cells were collected, pelleted and stained with BODIPY 493/503 (2 μg/mL; 1 h, 37°C, Cayman) as described elsewhere (8, 18), and analyzed by flow cytometry (Accuri C6^®^, Becton-Dickinson). Finally, cells were gated according to their size and granularity; BODIPY 493/503-derived signals were assessed in the FL-1 channel as previously described (12, 17).

### RT-qPCR for relative quantification of NPC mRNA

To evaluate the effects of *B. besnoiti* infection on host cellular NPC1 gene transcription, qPCR of *B. besnoiti*-infected cell was performed. BUVEC were cultivated in 25 cm^2^ tissue culture flasks (Greiner) and infected with *B. besnoiti* tachyzoites (MOI = 5:1). At 3, 6, 12 and 24 h p. i., infected host cells and non-infected controls were processed for RNA isolation by RNeasy kit (Qiagen), according to manufacturer’s instructions. Contaminating genomic DNA was removed by DNA digestion (10U DNase I, Thermo Scientific) following manufacturer’s instructions. The efficacy of genomic DNA digestion was confirmed by no-RT-controls in each RT-qPCR experiment. For cDNA synthesis, SuperScript IV (Invitrogen) was used in accordance with manufacturer’s instructions. The probes, labeled at the 5′-end with a reporter dye FAM (6-carboxyfluorescein) and at the 3′-end with the quencher dye TAMRA (6-carboxytetramethyl-rhodamine), were designed for NPC1 with the following sequences: *Bos taurus* NPC1 forward 5′-TCTGGAGATA AGAGATACAAC-3′; reverse 5′-CTGACATTGCCAAAGAAG-3′, and probe CACCAGAGCCATTGCCACAG. qPCR-based DNA amplification was conducted on a Rotor-Gene Q Thermocycler (Qiagen) in duplicates within a 10 μL total volume containing 400 nM forward and reverse primers, 200 nM probe, 10 ng cDNA, and 5 μL 2× PerfeCTa qPCR FastMix (Quanta Biosciences). The reaction conditions included an initial step at 95°C for 5 min, followed by 45 cycles at 94°C for 15 s, 60°C for 60 s, and 72°C for 30 s. Relative gene transcription quantification was performed using ΔΔCt method where GAPDH served as the reference housekeeping gene, as reported previously (8).

### Statistical analysis

Statistical analyses were carried out with the software GraphPad^®^ Prism 8 (version 8.4.3.). Data description included arithmetic mean ± standard deviation of five biological replicates. In addition, the non-parametric statistical Mann–Whitney test for comparison of two experimental conditions was applied. In cases of three or more conditions, Kruskal–Wallis test was used. Whenever global comparison by Kruskal–Wallis test indicated significance, *post hoc* multiple comparison tests were carried out by Dunn tests to compare test with control conditions. Outcomes of statistical tests were considered to indicate significant differences when *p* ≤ 0.05 (significance level).

## Results

### U18666A treatment inhibits *Besnoitia besnoiti* tachyzoite proliferation and induces dense vesicle accumulation

**Figure 1:**
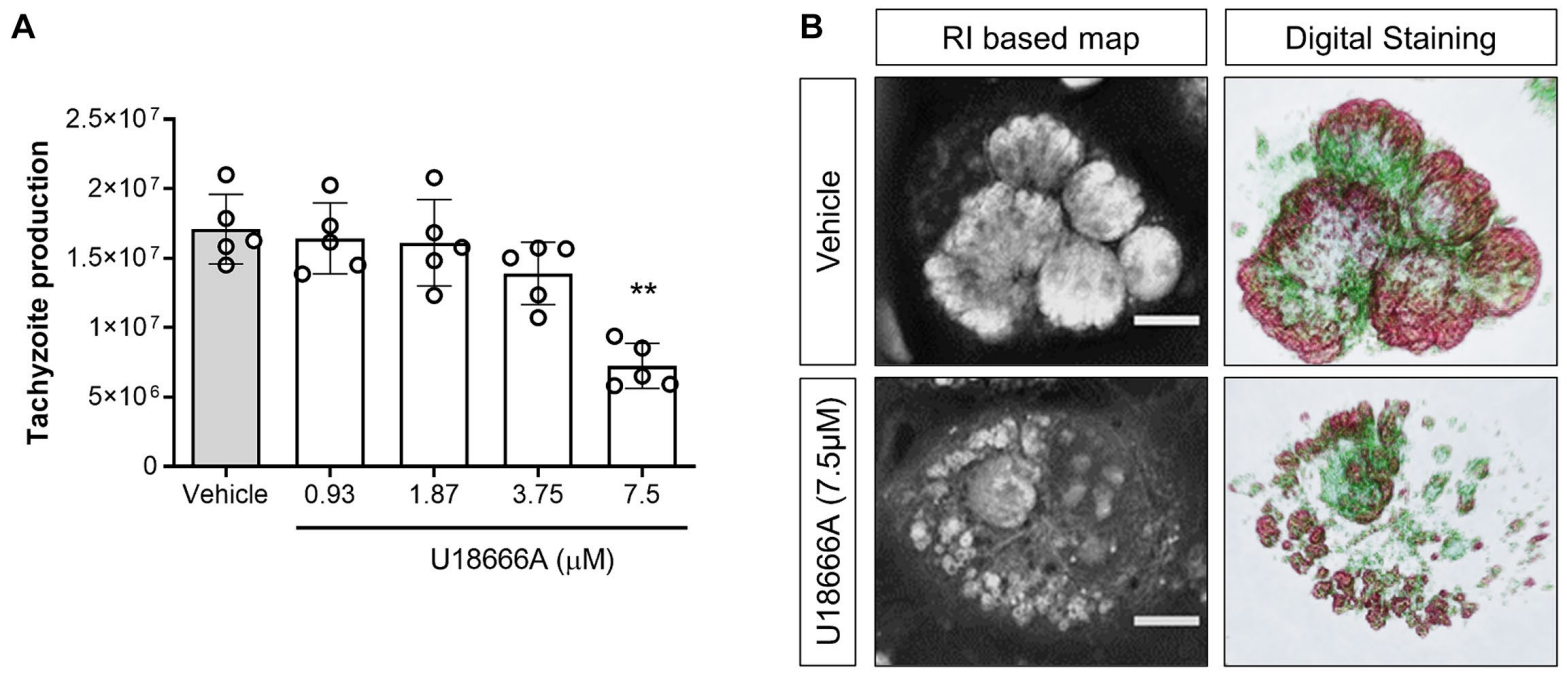
NPC1 inhibition reduces *B. besnoiti* intracellular proliferation. **(A)** BUVEC were treated with U18666A (0.93, 1.87, 3.75 and 7.5 μM) 48 h before *B. besnoiti* infection (MOI 1:5). At 48 h p. i., the number of tachyzoites present in cell culture supernatants were counted. **(B)** Exemplary illustration of live cell 3D holotomography and digital staining of vehicle- or U18666A (7.5 μM)-treated *B. besnoiti*-infected BUVEC at 24 h p. i. Scale bar represents 5 μm. Bars represent means of five biological replicates ± standard deviation. ^**^p ⩽ 0.01.

**Figure 2:**
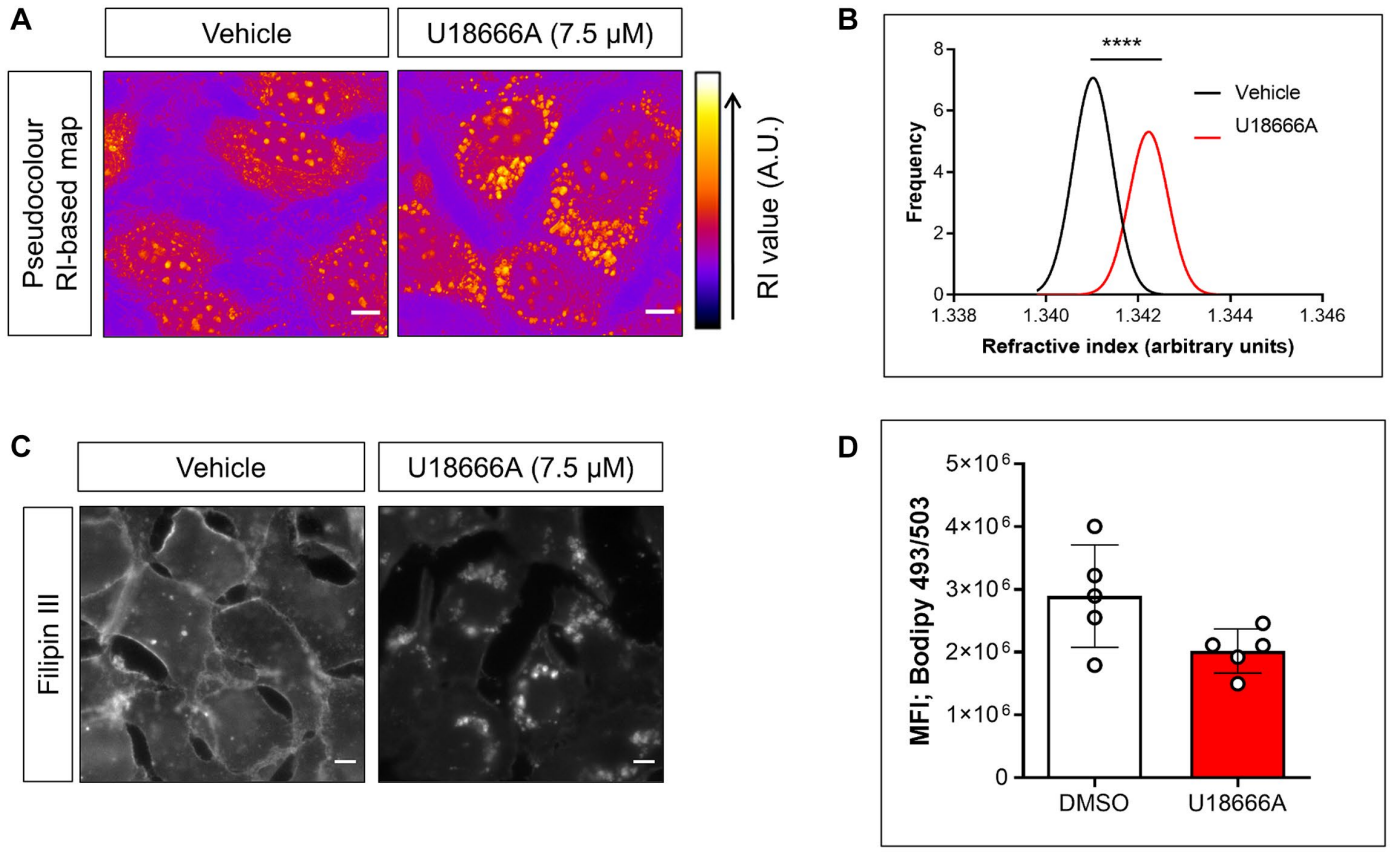
U18666A treatment affects subcellular cholesterol distribution and single cell RI without changing cellular neutral lipid content. **(A)** Live cell holotomography-deduced RI based images of vehicle- or U18666A (7.5 μM)-treated BUVEC depicted in pseudocolour. **(B)** Frequency histogram of single cell-based average RI of vehicle- or U18666A (7.5 μM)-treated cells. **(C)** Fillipin III-driven fluorescence signals of vehicle- or U18666A (7.5 μM)-treated BUVEC. **(D)** Flow cytometry-based quantification of BODIPY 493/503-derived fluorescence signals of vehicle- or U18666A (7.5 μM)-treated cells. Scale bar represents 5 μm. Bars represent means of five biological replicates ± standard deviation. ^****^p ⩽ 0.0001.

The participation of host NPC1 on *B. besnoiti* proliferation was determined by treatments with its specific inhibitor U18666A. Overall, pharmacological blockage of NPC1 by 7.5 μM U18666A treatments led to a significant reduction (*p* = 0.003) of *B. besnoiti* replication by 56.4 ± 12.96% (IC_50_: 10.42 μM ± 2.36; Figure 1A) at 48 h p. i. Likewise, live cell 3D holotomography of *B. besnoiti* meront development in U18666A-treated host cells showed less parasitophorous vacuoles (PV) each containing fewer tachyzoites, visibly affected in morphology at 24 h p. i., when compared with vehicle-treated host cells (Figure 1B). Additionally, U18666A treatments (7.5 μM) led to an accumulation of refractive-dense vesicles (average RI 1.358 ± 0.005; *n* = 100 vesicles) within the cytoplasm of infected and non-infected BUVEC (Figure 2A). In line, single cell analysis revealed an increase in RI average by 13.01% (*p* < 0.0001) driven by U18666A treatments (Figure 2B). Finally, no effect on *B. besnoiti* tachyzoite infectivity was observed after a 30 min treatment of free tachyzoites with U18666A at 7.5 μM (Supplementary Figure S1).

### U18666A treatment affects cholesterol distribution, without inducing neutral lipids accumulation

Given the effect observed by U18666A treatments on RI-based maps, its effect on cellular total cholesterol distribution and neutral lipid content was evaluated by filipin III and BODIPY 493/503 staining, respectively (22). Overall, an inhibitor (U18666A)-driven translocation of filipin III-derived cholesterol signals from cytoplasmic membranes into cytoplasmic vesicles was detected (Figure 2C). In contrast and reflecting cellular neutral lipid content, no statistical significant (*p* = 0.095) effect of U18666A treatments on BODIPY 493/503-derived total signal intensities was observed (Figure 2D) indicating an U18666A-mediated effect on subcellular cholesterol/neutral lipid distribution, but not on its total amount.

### The NPC1 gene is not modulated by *Besnoitia besnoiti* infection in BUVEC

**Figure 3:**
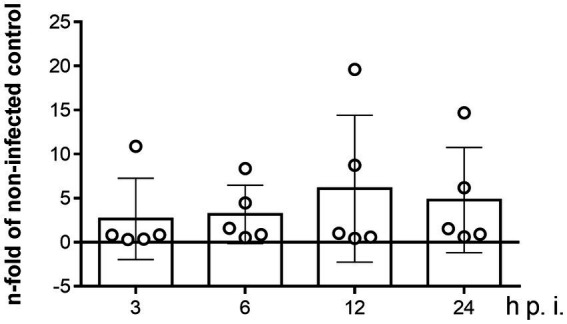
Kinetics of NPC1 gene transcription in *B. besnoiti*-infected BUVEC. BUVEC were infected with *B. besnoiti*-tachyzoites and monitored for NPC1 gene transcription over time via qPCR. Bars represent the means of five biological replicates ± standard deviation. Significance level: *p* < 0.05.

Since apicomplexan parasites are capable of modulating host gene expression to ensure their obligate intracellular replication metabolic requirements, the effect of *B. besnoiti* infection on NPC1 mRNA expression in BUVEC was evaluated over time by RT-qPCR. Even though NPC1 gene transcription revealed slightly enhanced at 12 and 24 h p. i. in single endothelial cell isolates (Figure 3), no significant *B. besnoiti* infection-driven changes in NPC1 gene expression were stated.

## Discussion

Considering that apicomplexan parasites are auxotrophic for cholesterol, they rely on host cellular sterol sources to satisfy their molecular requirements for obligate intracellular proliferation (9). In this context, LDL-based cholesterol uptake represents a typical cholesterol source for obligate intracellular apicomplexan species. Overall, based on the key role of LDL endocytic routes, here we explored the participation of NPC1 in *B. besnoiti* tachyzoite proliferation, thereby presenting the first report on this sterol carrier for *B. besnoiti* infections in primary bovine endothelial cells. In detail, a pivotal role of NPC1 for *B. besnoiti* replication was confirmed since pharmacological NPC1 blockage by U18666A (7.5 μM) treatments significantly inhibited tachyzoite replication in BUVEC, with an overall reduction of 56.4%. In line, former reports already indicated a principle antimicrobial capacity of NPC1 blockage for different virus infections like hepatitis C virus, Ebola virus, Dengue virus, Pseudorabies virus, and feline coronavirus (24–27). Referring to apicomplexan parasites, NPC1-related data are relatively scarce. Nonetheless, prior work showed that U18666A treatments (2.3 μM) reduced *T. gondii* replication by 72% in CHO cells (10). Likewise, NPC1 blockage also interfered with *E. bovis* macromeront development in BUVEC (18). Moreover, U18666A treatments not only impaired *Plasmodium berghei* schizont development in Heh7 cells, MEF cells and primary hepatocytes, but also reduced parasite burden in mice *in vivo* (17), suggesting a general, NPC1-related mechanism in apicomplexan intracellular replication. Interestingly, current findings demonstrated only a partial effect of NCP1 inhibition on *B. besnoiti* development thereby implying alternative routes of cholesterol acquisition. In agreement, coccidia-driven upregulation of *de novo* cholesterol biosynthesis and cholesterol uptake from extracellular sources by other receptors like OLR-1 or SR-BI have been proposed (6, 8). Mechanistically, U18666A treatments prevent the exit of cleaved cholesteryl esters from late endosomes, thus driving free cholesterol accumulation in these organelles (20). In this context, we first assessed NPC1 blockage in U18666A-treated BUVEC by 3D live cell holotomography, permitting a functional insight into live cell morphology through refractive properties assessment (28). Here, a significant accumulation of refractive-dense vesicles in the cytoplasm of U18666A-treated BUVEC was detected. The morphological characteristics of these vesicles matched with a prior report on U18666A-treated HeLa cells where these structures were identified as late endosomes (28). Moreover, the impact of NPC1 blockage on cholesterol transport in primary bovine endothelial cells was confirmed by filipin III-based staining of cholesterol. Hence, U18666A treatments caused a subcellular redistribution of filipin III-based signals from evenly stained membranes in non-treated cells into visible cytoplasmic vesicles in treated BUVEC. Physiologically, intracellular cholesterol levels are tightly regulated by feedback mechanisms that operate at transcriptional levels. The content of cholesterol/cholesteryl esters triggers the upregulation of molecules that mediate their efflux (i.e., ABCA1) (29). In line, FACS-based analyses showed no changes in total BODIPY 493/503-derived signals in U18666A-treated BUVEC, indicating that NPC1 blockage does not induce changes in total cholesterol/cholesteryl ester content but exclusively drives subcellular compartmentalization of these sterols which then are unavailable for cytoplasmic *B. besnoiti* stages consumption within PV.

Host cell gene modulation is a mechanism widely reported to support the successful replication of other apicomplexan parasites (30, 31). Nonetheless, NPC1 regulation in apicomplexan-parasitized host cells is not fully understood. However, it is linked to a sterol-regulatory binding protein (SRBP)-dependent mechanism, with lipid availability and downstream cholesterol metabolite abundance regulating NPC1 gene expression (32, 33). Despite that, NPC1 overexpression increased sterol esterification in CHO cells (34). Therefore, we also analysed the capacity of *B. besnoiti* tachyzoites to modulate NPC1 gene expression over time. Unexpectedly, no infection-driven changes on NPC1 on the transcriptional level were noted. This contrasts with findings on the closely related parasite *N. caninum*, in which transcriptomic analysis of infected trophoblastic cells revealed an upregulation of this gene at 8 h p. i (30, 35).

In conclusion, the current findings confirm a participation of NPC1 in *B. besnoiti* merogonic proliferation in BUVEC, thereby highlighting the role of this host protein in apicomplexan biology. Further analyses are needed to evaluate whether the blockage of cholesterol redistribution via NPC1 inhibition may represent a potential therapeutical target not only against cyst-forming-but also against non-cyst-forming-coccidian parasites.

## Data availability statement

The raw data supporting the conclusions of this article will be made available by the authors, without undue reservation.

## Ethics statement

Ethical approval was not required for the studies on animals in accordance with the local legislation and institutional requirements because only commercially available established cell lines were used.

## Author contributions

CL: Conceptualization, Formal analysis, Investigation, Writing – original draft, Writing – review & editing. CH: Project administration, Resources, Visualization, Writing – review & editing. AT: Conceptualization Funding acquisition, Resources, Supervision, Writing – review & editing. LS: Conceptualization Formal analysis, Investigation, Supervision, Writing – review & editing, Writing – original draft.

## Supplementary material

SUPPLEMENTARY FIGURE S1 U18666A does not affect *B. besnoiti* infectivity in BUVEC. *B. besnoiti* tachyzoites were exposed to either the vehicle or U18666A for 30 min and then allowed to infect BUVEC monolayers. Bars represent the means of five biological replicates ± standard deviation.
