# Supplementary figures and images for: Besnoitia besnoiti tachyzoite replication in bovine primary endothelial cells relies on host Niemann–Pick type C protein 1 for cholesterol acquisition
Source: Front Vet Sci. 2024 Aug 9;11:1454855. doi: 10.3389/fvets.2024.1454855 (PMC11341383; doi:10.3389/fvets.2024.1454855)

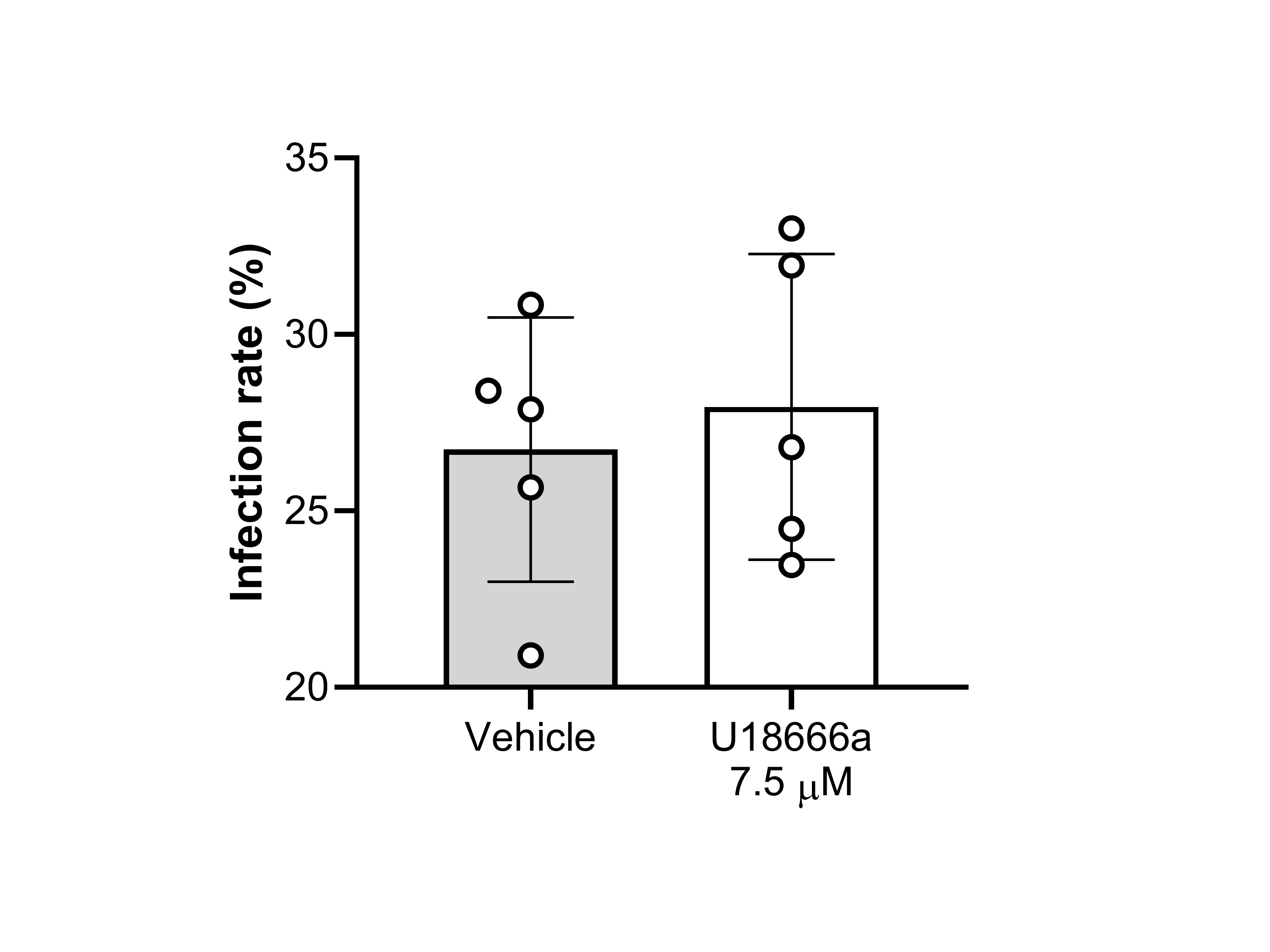

Supplement: SUPPLEMENTARY FIGURE S1 — U18666A does not affect B. besnoiti infectivity in BUVEC. B. besnoiti tachyzoites were exposed to either the vehicle or U18666A for 30 min and then allowed to infect BUVEC monolayers. Bars represent the means of five biological replicates ± standard deviation. [file Image_1.TIF]
